# Combining Personality Traits with Traditional Risk Factors for Coronary Stenosis: An Artificial Neural Networks Solution in Patients with Computed Tomography Detected Coronary Artery Disease

**DOI:** 10.1155/2013/814967

**Published:** 2013-10-03

**Authors:** Angelo Compare, Enzo Grossi, Massimo Buscema, Cristina Zarbo, Xia Mao, Francesco Faletra, Elena Pasotti, Tiziano Moccetti, Paula M. C. Mommersteeg, Angelo Auricchio

**Affiliations:** ^1^University of Bergamo, Piazzale S. Agostino 2, P.O. Box 24129, Bergamo, Italy; ^2^Villa Santa Maria Institute, IV Novembre, P.O. Box 22038, Tavernerio, Italy; ^3^Semeion, Research Centre of Sciences of Communication, Via Sersale 117, P.O. Box 00128, Rome, Italy; ^4^Department of Mathematical and Statistical Sciences, University of Colorado at Denver, P.O. Box 173364, Denver, CO, USA; ^5^School of Electronic and Information Engineering, Beihang University, Xueyuan Road No. 37, Haidian District, Beijing, China; ^6^Division of Cardiology, Cardiocentro Lugano CH-6900, Switzerland; ^7^Center of Research on Psychology in Somatic Diseases, CoRPS, Tilburg University, Warandelaan 2, P.O. Box 90153, 5000 LE Tilburg, The Netherlands

## Abstract

*Background*. Coronary artery disease (CAD) is a complex, multifactorial disease in which personality seems to play a role but with no definition in combination with other risk factors. *Objective*. To explore the nonlinear and simultaneous pathways between traditional and personality traits risk factors and coronary stenosis by Artificial Neural Networks (ANN) data mining analysis. *Method*. Seventy-five subjects were examined for traditional cardiac risk factors and personality traits. Analyses were based on a new data mining method using a particular artificial adaptive system, the autocontractive map (AutoCM). *Results*. Several traditional Cardiovascular Risk Factors (CRF) present significant relations with coronary artery plaque (CAP) presence or severity. Moreover, anger turns out to be the main factor of personality for CAP in connection with numbers of traditional risk factors. Hidden connection map showed that anger, hostility, and the Type D personality subscale social inhibition are the core factors related to the traditional cardiovascular risk factors (CRF) specifically by hypertension. *Discussion*. This study shows a nonlinear and simultaneous pathway between traditional risk factors and personality traits associated with coronary stenosis in CAD patients without history of cardiovascular disease. In particular, anger seems to be the main personality factor for CAP in addition to traditional risk factors.

## 1. Introduction

Coronary artery disease (CAD) is a complex, multifactorial disease in which genetic predisposition, lifestyle, and environmental risk factors play a key role, the combination of which is not known.

Studies on traditional cardiac risk factors have shown that older age, higher body mass index, male gender, diabetes, hypertension, and dyslipidemia increase the likelihood of the coronary artery plaque (CAP) burden and, moreover, these risk factors are directly related to 10-year risk of fatal cardiovascular events (CVE) [1].

Framingham Heart Study models [2] and the SCORE model [3] are predictive risk charts and among the more extensively used ones in cardiovascular disease prevention. These models are based on logistic regression [4] or Cox proportional hazards [5] predictive algorithms. However, weaknesses in the calibration and discrimination have been acknowledged which affect the reliability of these risk prediction tools [6]. Limitations could be due to the existence of unknown risk factors, incidence rates of the disease, and factors that are difficult to reproduce in every day. Although algorithms for cardiovascular risk assessment are accurate on a population level, they may fall short at an individual level, most likely due to wide confidence intervals of risk algorithms and inability to fully capture dynamic process [7]. Indeed, a significant number of CVE were found to occur either in the absence of known risk factors or in the presence of moderate risks when an aggressive treatment strategy would not be indicated. Findings show that 18% of subjects expected to be at low risk of CVE (no known risk factor) had evidence of CAP, while in about 10% of patients who had three risks or more factors normal coronary arteries were found [1]. Log linear models, most often used, are probably inadequate since they cannot accommodate many covariates and other correlated covariates, such as age, which should be used as a “risk condition” but not as a risk factor. Furthermore, the linearity of these models for CAD risk prediction should be further evaluated. A review of the current literature shows few attempts to use the Artificial Neural Networks (ANN) approach to predict cardiovascular risk. In the Prospective Cardiovascular Munster Study (PROCAM), the comparison between ANN and classical logistic regression to predict future cardiovascular events, despite neural networks and multiple logistic models, was run with dissimilar covariates, and the superior performance of ROC area under the curve (AUC) with training ANN versus logistic regression model was however clear-cut, with values, respectively, equal to 0.897 and 0.840 [8]. In addition, a subsequent study showed that CHD mortality prediction by training AAN or multiple logistic function had similar (0.669–0.699) results receiver operating characteristic AUC [9].

There is growing evidence that personality traits may contribute directly, by physiological mechanisms, and indirectly, by unhealthy life style, to CAD. In particular findings show that anger, hostility [10], and Type D personality [11] traits may play crucial roles in the pathogenesis of CAD. These data may reinforce the hypothesis that personality traits may have a potential role in the development of CAP. Therefore, a better understanding of the combination between traditional and personality traits appears to be crucial for refining the risk profile for CAD.

The analysis of the combination between traditional and personality traits for CAD risk may require to explore nonlinear and simultaneous combination between the variables [12, 13]. Traditional statistical approaches may not be appropriate or powerful enough to address this challenge so that it requires innovative data mining analysis based on Artificial Neural Networks (ANN) algorithms. Although ANN algorithms have been applied in predicting long-term coronary heart disease mortality [8, 9], no research has investigated nonlinear and simultaneous combination between traditional risk factors and personality traits for detecting presence of coronary stenosis.

In this study, we sought to explore the nonlinear and simultaneous pathways between traditional risk factors and personality traits for detecting coronary stenosis by using ANN data mining analysis.

## 2. Methods

### 2.1. Sample and Procedure

In the period between October 2009 to July 2010 all subjects who received 64-slice Computed Tomography Coronary Angiography (CTCA), at the Division of Cardiology of Fondazione Cardiocentro Ticino, Lugano, Switzerland, were informed about the study during their first admission visit.

Indications for performing a CTCA were chest pain syndrome, shortness of breath, syncope, or equivocal stress testing including exercise ECG, myocardial perfusion imaging, or stress echocardiography unable to definitively rule out/rule in significant coronary artery disease. Exclusion criteria for performing CTCA were renal insufficiency (serum creatinine 120 mol/L), contraindications to the administration of iodinated contrast, pregnancy, acute coronary syndromes, and ventricular and/or supraventricular arrhythmias. All individuals gave informed consent, and the study protocol was approved by the institutional review board. The general flow of study has been presented in a previous report [14]. Exclusion criteria were “history of coronary artery disease” or “acute coronary syndrome,” having a psychiatric disorder, or being treated with psychotropic medication. Inclusion criteria were chest pain syndrome, shortness of breath, syncope, or equivocal stress testing including exercise ECG, myocardial perfusion imaging, or stress echocardiography unable to definitively rule out/rule in significant coronary artery disease. All seventy-five subjects who agreed to participate in the study provided written informed consent. Psychological questionnaires were filled in the hospital two days before the CTCA scan. Participants met individually with a certified clinical psychologist for a short clinical interview and filled out the questionnaires. For each individual, medical history and detailed physical examination were obtained by patient medical record. Systolic and diastolic blood pressures were measured in sitting position after 5 minutes of rest using an oscillometric validated device [15].

### 2.2. Cardiac Risk Factors

The following traditional cardiac risk factors were examined: hypertension: arterial blood pressure ≥ 140/90 mm Hg or taking antihypertensive medications [16]; diabetes: nonfasting plasma glucose concentration of at least 200 mg/dL (11.1 mmol/L), or fasting plasma glucose level of at least 126 mg/dL (7.0 mmol/L), or being treated with antidiabetic medication; overweight: body mass index (BMI) (calculated as weight divided by height squared) ≥27 kg/m^2^ (WHO); dyslipidemia: total serum cholesterol level is higher than 240 mg/dL or a serum triglyceride level is 200 mg/dL or more (or both) or use of a lipid-lowering agent; smoking: at least one cigarette per day or quit smoking during the previous year; family history of CAD: a first degree or second degree relative with premature cardiovascular disease (age ≤ 55 years).

### 2.3. Coronary Stenosis Assessment

CTCA assessment of the coronary arteries was done with the bolus tracking technique (SmartPrep), using a 64-slice CT scanner (LightSpeed VCT, GE Healthcare, Milwaukee, WI, USA). A detailed description of the procedure is provided by Faletra and colleagues [1]. Image data sets were reconstructed immediately after the scan. Two experienced observers with knowledge of the individual's clinical history and indications for patient referral evaluated CTCA in a joint reading manner. Coronary obstructions were evaluated by visual assessment comparing the luminal diameter of the segment exhibiting the obstruction to the luminal diameter of the most normal appearing site immediately proximal to the plaque. Coronary lumen narrowing was used to detect the stenosis degree and graded semiquantitatively and classified as normal (no plaque or up to 30% of coronary lumen diameter), mild (up to 50% of coronary lumen diameter), moderate (51–70%), and severe (>70%). In case of discordance between the two readers, they proceeded with a consensual reevaluation.

### 2.4. Personality Trait Measures

#### 2.4.1. Hostility

Hostility was assessed using the 27-item version of the Cook-Medley Hostility Scale [17] (Barefoot et al., 1989) which is thought to reflect the cognitive, behavioural, and mood components of hostility. Items are scored on a dichotomized scale (1 = True, 0 = False), and the total score reflects tendency to express cynicism, hostile affect, and aggressive response [18, 19]. Cronbach's alpha was 0.87 in the present study. To investigate the prevalence of trait hostility within discrete categories of CAP severity, in addition to the continuous measure, a psychometric cut-off value of *T*-score value ≥65 was used. On the basis of MMPI manual [20, 21], the *T*-score value was calculated to adjust to the score obtained for demographic information by standardization tables. A *T*-score value ≥65 corresponds to a score greater than the ninety-two percentile, which equates to a higher level of “moderate” [22].

#### 2.4.2. Anger

Anger was measured with the 16-item Anger Scale of the MMPI-2 (MMPI-ANG) [20, 21]. MMPI-2 ANG Scale is a reliable index of predisposition to the external expression of anger and this scale was found to be associated with CHD incidence and myocardial infarction in large prospective studies.

Items are scored on a dichotomized scale (1 = true, 0 = false), and the total high score reflects frequent and intense anger, feeling frustrated, being quick-tempered, and being impulsive and prone to interpersonal problems [23]. In the present study Cronbach's alpha was 0.79. To investigate the prevalence of trait anger within discrete categories of CAP severity, in addition to the continuous measure, a psychometric cut-off *T*-value score of ≥65 was used. On the base of MMPI manual [20, 21], the *T*-score value is calculated to adjust to the score obtained for demographic information by standardization tables. A *T*-score value ≥65 corresponds to a score greater than the ninety-two percentile, which equates to a higher level of “moderate.”

#### 2.4.3. Type D Personality

Type D personality was measured with the 14-item Type D Personality Scale (DS14) [24]. Type D personality is characterized by the tendency to experience negative emotions and no to express these emotions in social interactions. It consists of 2 subscales: negative affectivity (NA) and social inhibition (SI). A score of 10 or more on both subscales denotes those with Type D personality. In the present study the internal consistency, calculated by Cronbach's alpha, for NA and SI subscales was 0.89 and 0.91, respectively. As has recently been suggested [25], in addition to the conventional categorisation, Type D can be considered as a dimensional construct by multiplying the NA and SI subscale totals as well as using continuous scores on both subscales.

### 2.5. Statistical Analysis

Investigation of nonlinear and simultaneous pathways between traditional and personality traits risk factors leading to coronary stenosis has been achieved through a new data mining method, based on a particular artificial adaptive system, the autocontractive map (AutoCM). AutoCM is a technique to compute the association of strength of each variable with all other variables in any dataset (i.e., in terms of many-to-many rather than dyadic associations). The architecture and mathematics of AutoCM are described elsewhere [26, 27]. AutoCM is a data mining tool based on an artificial neural networks (ANN) [26], which is especially effective in highlighting any kind of consistent patterns, systematic relationships, hidden trends, and associations among variables, as well as in acute myocardial infarction risk analysis study [28]. The weights determined by AutoCM after the training phase permit a direct interpretation. Specifically, they are proportional to the strength of many-to-many associations across all variables. This allows a further, useful processing: association strengths may be easily visualized by transforming weights into physical distances. Such a “translation” proceeds in an intuitive way: variables whose connection weights are higher get relatively nearer and vice versa. By applying a mathematical filter such as the minimum spanning tree to the matrix of distances, a graph, named “semantic connectivity map,” is generated, which has been tested in the medical field [26]. This representation allows a visual mapping of the complex web of connection schemes among variables. The AutoCM matrix of connections preserves nonlinear associations among variables, while at the same time captures elusive connection schemes among clusters that are often overlooked by cluster analyses and highlights complex similarities among variables. On this data set, AutoCM algorithm was applied to dichotomized variables on complete group including both people with CAP presence and absence or only the CAP presence group. Variables enclosed into analysis are presented in Table 1. Results of AutoCM algorithm analysis are depicted in by a “semantic connectivity map” and “analysis of hidden connections through the Maximmally Regular Graph algorithm [26, 27].” The stability of the statistical method was verified by a validation protocol [28]. A principal component analysis of 1st and 2nd component has been applied to the same data set as a linear benchmarking, using the MATLAB package.

## 3. Results

### 3.1. Nonlinear and Simultaneous Pathways between Traditional and Personality Traits Risk Factors

**Figure 1:**
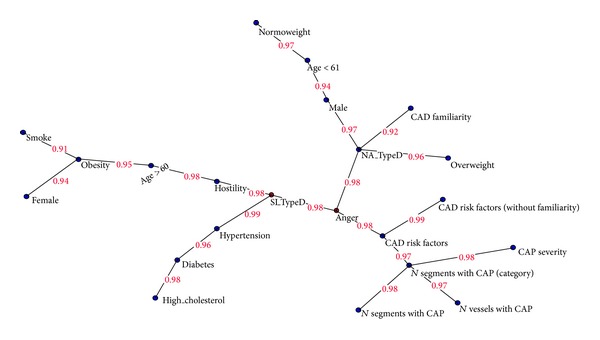
Semantic connectivity map. Note: semantic connectivity map of the variables under study in the study group with AutoCM system. The values on the arcs of the graph indicate the strength of the connection, measured on a scale ranging from zero to 1.

Figure 1 shows a semantic connectivity map (SCM) performed by AutoCM analysis for all factors. All variables showed a stability index very close to full agreement, and the mean stability index of the variables from the validation protocol was 98.49%, with a variance of 0.0003. The SCM describes the correlation between cardiovascular risk factors and personality traits, providing insight into pattern and strength of association.

The SCM confirms the importance of the number of traditional risk factors, taken together, for CAP. Anger is the main personality factor for CAP in numbers of traditional risk factors. Furthermore, based on these findings anger appears to be a hub for two-risk profiles associated with the two components of Type D personality: negative affectivity (NA) and social inhibition (SI).

The SI component of Type D acts as a hub on two branches: (a) hypertension, diabetes, and cholesterol; (b) hostility, age over 60, obesity, smoking, and female sex. In fact, data suggest that women older than 60 years old who smoke are more likely to be overweight.

The NA component of Type D instead acts as a hub to three branches: (a) overweight; (b) CAD family history; (c) male gender, age less than 61, and normal weight. In particular, SCM shows that negative affectivity has a strong relation with being overweight. However, male gender presents a strong relation with normal weight.

**Figure 2:**
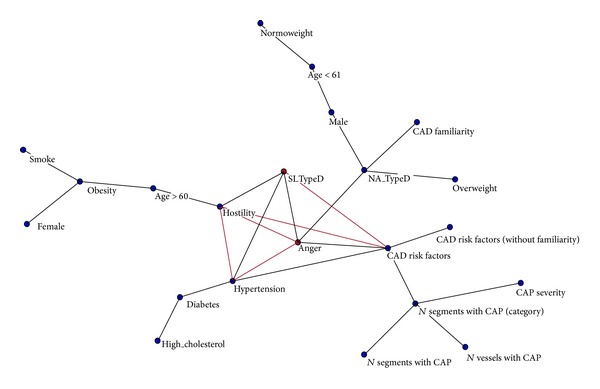
Analysis of the hidden connections through Maximally Regular Graph.

The analysis of the hidden connections (Figure 2) shows a circular connection between a number of traditional risk factors with anger, social inhibition, hostility, and hypertension. The hidden connection map shows that anger, hostility, social inhibition, and hypertension are the main factors related to traditional cardiovascular risk factors (CRF), which in turn are significantly associated to CAP.

**Figure 3:**
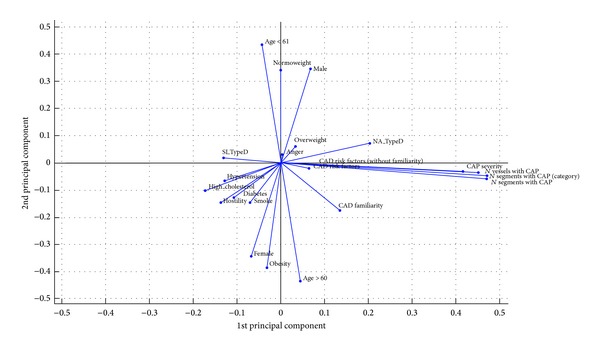
Principal component analysis; first and second components.

Results of PCA represent a benchmark for the adaptive data mining based on ANN. After analysis of the variance explained by all the components, the first two components which explain almost 50% of the variance were selected. The variables related to the CAP are correlated to each other and associated with the 2nd component, while other variables are weakly associated with the 2nd component (Figure 3).

## 4. Discussion

This study shows nonlinear and simultaneous pathways between traditional and personality traits risk factors in relation to coronary stenosis in asymptomatic subjects. In particular, results show that anger is the main personality factor for CAP in numbers of traditional risk factors and is, moreover, significantly related to the two components of the Type D personality: social inhibition and negative affectivity.

These data confirm the existing literature on this topic, that anger is considered to be an independent risk factor for coronary heart disease (CHD), although there are mixed findings [29, 30]. In the past, anger was studied jointly with hostility as a unitary cardiac risk factor. It was difficult to maintain a net distinction because anger also implies cognitive and relational factors and, like hostility, it implies the tendency to act in an aggressive mode [31]. The literature has shown that anger is associated with other risk factors of cardiac disease through some direct (biological) and indirect (behavioral) processes. Anger increases catecholamine discharge, causes elevation of blood pressure and heart rate, potentiates vasospasm, and enhances hemodynamic stress, which may in turn disrupt a vulnerable plaque, trigger hemostasis and vasoconstriction, and result in occlusive ischemic sequelae [32, 33]. Moreover, chronic and repeated exposure to anger-provoking stressful situations and frequent overt anger expression are associated with repeated bouts of HPA, sympathoadrenomedullary and endogenous opioid system activity [34]. Repeated activation may contribute to susceptibility and increases allostatic load across these systems [35]. Studies underscore the indirect relation between anger and cardiac disease, showing that high levels of anger are likely to increase adverse behaviors. A style of overt anger expression and a high level of anger experience may contribute to various maladaptive behavioral responses, including a greater likelihood of substance abuse such as smoking and alcohol use [36, 37].

Anger has been related to Type D personality. Type D individuals are characterized by an increased tendency to experience negative emotions, such as depressed mood, anxiety, anger, and hostile feelings, and to inhibit them in social interactions [38]. Many studies have shown that this personality trait is an independent risk factor for recurrent cardiovascular events and indicative of poor somatic and psychological health among patients with established coronary heart disease (CHD) [39–41]. Consistent with the existing literature, the present study shows a significant relation of social inhibition a typical cluster of Type D personality, characterized by expecting negative reactions from others and tending to be socially isolated, and hypertension. Findings from other studies suggest that social inhibition influences cardiac prognosis through potential pathways such as cardiovascular reactivity to stress [42–44], reduced heart rate variability [45], increased inflammation [46], and blood pressure reactivity [44]. Type D personality related behavior was linked to physiological hyperresponsivity [47], leading to hypertension in which reactivity is thought to lead to increased peripheral resistance which contributes to elevated blood pressure over time [48]. Hyperreactivity could lead to heart disease by causing injury to the endothelial lining of the arteries, thereby promoting the accumulation of plaque, which, over time, can lead to acute events such as thrombosis or ischemia [49]. Social inhibition may also impede communication between patient and physician, with potential damage to health [50]. PCA is mathematically defined as an orthogonal linear transformation that remaps possibly correlated data into a new coordinate system of uncorrelated variables, such that the largest possible amount of variance is mapped onto the first (uncorrelated) variable (called the first principal component) and the second largest amount of (residual) variance is mapped onto the second coordinate. In this way, by discarding lower-order principal components, the information loss is relatively small, as most of the variance of the phenomenon is gathered in the higher-order components. Overall, the variance explained by the first two components of the PCA for our data set is about 50%: there is, thus, a nonnegligible informational loss, whereas the AutoCM technique basically captures all of the available information.

Results of this study show a significant relation between negative affectivity and overweight. The previous literature confirms a relation between being overweight and negative affect defining emotional eating as the tendency to overeat in response to negative emotions such as anxiety or irritability [51]. Therefore, personality traits may also influence eating habits and lead to overeat/overweight, a leading cause of cardiac disease. Emotions such as depression, anxiety, and anger were the most frequently reported precipitants of emotional eating. Since having obesity often coincides with depressive symptoms, the food intake of people with obesity could be explained by a coping strategy used to reduce feelings of negative affect. Obesity and overweight, in turn, are well-know leading causes of cardiac disease that may increase the risk of CAD and adverse cardiovascular events in several pathophysiological pathways. Obesity may reduce insulin sensitivity, enhance free fatty acid turnover, increase basal sympathetic tone, induce a hypercoagulable state, and promote systemic inflammation [52].

### 4.1. Strengths and Limitations

Our study has a number of limitations: first of all, the sample size is limited which does not allow firm conclusions. However, the use of ANN allowed us to minimize the bias due to sample size [7, 26, 53]. A second limitation is determined by the mode of measurement of personality traits based on self-report questionnaire that presents bias due to subjectivity. The main strength of this study is given by the method of data analysis, the ANN, which allows to analyze the nonlinear relationship between the variables. The PCA was unable to find relevant associations between personality traits, cardiac risk factors, and coronary stenosis due to the poor linear correlation among them.

### 4.2. Clinical Consideration

Early identification of cardiovascular patients who are characterized by an unfavorable clustering of personality traits, such as anger and Type D personality, could aid in improving their prognosis and quality of life. This could lead to treatment benefits in the field of psychocardiology [54–57]. A reliable risk estimation is an important tool in primary and secondary prevention as it can be used to expedite the initiation of lifestyle changes or the use of an appropriate therapeutic intervention or both. Moreover, in addition to focusing on specific psychological risk factors, there is a need to adopt a personality approach in the early identification of those cardiac patients who are at risk of emotional stress-related cardiac events.

To conclude, ANN analysis allows to highlight the complexity of the connections between personality traits, traditional cardiac risk factors, and coronary stenosis, which are part of a more complete risk profile in patients with CAD.

## Figures and Tables

**Table 1:** Group description.

	*N* (or mean) *N* = 75	% (or SD)
Age	62.45	9.35
Male gender	42	56
Marital status		
Married	40	53.3
Single	35	46.6
Personality traits classification		
Hostility	50	66.7
NA TypeD	45	60.0
SI TypeD	48	64.0
Anger	53	70.7
CAD risk factors		
Dyslipidemia	22	29.3
Hypertension	43	57.3
Smoke	18	24.0
Diabetes	29	38.7
CAD familiarity	21	28.0
Overweight^*∧*^		
Normal	19	25.3
Overweight	33	44.0
Obese	23	30.7
*N* CAD risk factors		
0	10	13.3
1	18	24.0
2	19	25.3
3	28	37.3
*N* CAD risk factors°		
0	13	17.3
1	20	26.7
2	21	28.0
3	21	28.0
CAP severity*		
Normal	18	24.0
Mild	31	41.3
Moderate	8	10.7
Severe	18	24.0
*N* vessels with CAP		
0	18	24.0
1	20	26.7
2	16	21.3
3	18	24.0
4	3	4.0
*N* segments with CAP		
0	18	24.0
1	19	25.3
2	5	6.7
3	7	9.3
4	14	18.7
5	7	9.3
6	5	6.7
*N* segments with CAP (category)^§^		
1	18	24.0
2	19	25.3
3	12	16.0
4	26	34.7

CAP: coronary artery plaque; CAD: coronary artery disease.

^∧^WHO Criteria BMI classification: 0 = <25 normal; 1 = 25–30.0 overweight; 2 = 30–35 obese.

*Normal = 0%–≤30%; mild = 31%–50%; moderate = 51%–70%; severe = >70%.

°CAD risk factors (without familiarity).

^§^2 = 1; 3 = 2-3; 4 = 4–19.

## Appendix

### The AutoCM System

The AutoCM learning equations, the specific mathematics linked to the “contractive factor”, and the association to minimum spanning tree (MST) algorithm were elsewhere described [53]. The specificity of AutoCM algorithm is to minimize a complex cost function with respect to the traditional ones as follows.

(1) Traditional minimization cost function:
  (A.1) $$\begin{array}{c} E=\mathrm{Min}\left\{\sum_{i}^{N}\sum_{j}^{N}\sum_{q}^{M}x_{i}^{q}\cdot x_{j}^{q}\cdot \sigma_{i,j}\right\};\;\sigma_{i,j}>0. \end{array}$$
(2) AutoCM minimization cost function:
  *E* = *Min*⁡⁡{∑_*i*_^*N*^∑_*j*≠*i*_^*N*^∑_*k*≠*j*≠*i*_^*N*^∑_*q*_^*M*^ *x*_*i*_^*q*^ · *x*_*j*_^*q*^ · *x*_*k*_^*q*^ · *A*_*i*,*j*_ · *A*_*i*,*k*_};
  *x*_*i*_^*q*^ = value of *i*th input unit *q*th pattern;
  *A*_*i*,*j*_ = 1 − (*w*_*i*,*j*_/*C*); *A*_*i*,*k*_ = 1 − (*w*_*i*,*k*_/*C*);
  *w*_*i*,*j*_ < *C*; *w*_*i*,*k*_ < *C*.
  *i*, *j*, *k* = indices for the variables—columns;
  *q* = index for the patterns—rows;
  *N* = number of variables (columns);
  *M* = number of patterns (rows).

The traditional minimization includes only second-order effects, while the AutoCM also considers the third-order effects. Thus, AutoCM algorithm is able to discover variables similarities completely embedded into the dataset and is invisibles to the other classic tools.
